# Noninvasive Urinary Biomarkers for Obesity-Related Metabolic Diseases: Diagnostic Applications and Future Directions

**DOI:** 10.3390/biom15050633

**Published:** 2025-04-28

**Authors:** Shumin Zhan, Xuelian Zhou, Junfen Fu

**Affiliations:** Department of Endocrinology, Children’s Hospital, Zhejiang University School of Medicine, National Clinical Research Center for Child Health, Hangzhou 310051, China

**Keywords:** obesity-related metabolic diseases, urinary biomarkers, noninvasive diagnostics, type 2 diabetes, netabolic syndrome, metabolic-associated fatty liver disease, hypertension

## Abstract

Obesity-related metabolic diseases include conditions linked to obesity, such as type 2 diabetes, hypertension, steatotic liver disease, and polycystic ovary syndrome. These disorders are primarily caused by insulin resistance, chronic inflammation, and excessive fat accumulation. They represent significant health challenges and often remain asymptomatic during their early stages. Traditional diagnostic tools, including blood glucose, lipid levels, blood pressure, and uric acid measurements, provide valuable insights but fall short of fully capturing the complexity of metabolic dysfunction. Consequently, there is a growing need for noninvasive, easily accessible biomarkers, especially those found in urine, to enable more accurate, sensitive, and patient-friendly diagnostic methods. Urine, with its diverse range of metabolites that reflect the body’s metabolic changes, is an ideal sample for early detection. Recent advancements in urine metabolomics and proteomics have highlighted the potential of urinary biomarkers for diagnosing obesity-related metabolic diseases. Despite challenges such as the need for standardized detection techniques and clinical validation, the integration of artificial intelligence and multi-omics approaches holds significant promise for enhancing diagnostic accuracy and advancing disease management strategies.

## 1. Introduction

### 1.1. Obesity-Related Metabolic Diseases and Current Diagnostic Challenges

In 2022, more than 1 billion people worldwide were living with obesity [1]. Obesity-related metabolic diseases, including type 2 diabetes, hypertension, and fatty liver disease, arise primarily from insulin resistance, chronic inflammation, and excessive fat accumulation, collectively imposing a major health burden. While these comorbidities can also occur in individuals without obesity, identifying them early in obese individuals is essential for effective intervention and the prevention of further complications.

The diagnosis of overweight and obesity is traditionally based on measurements of weight and height, with the body mass index (BMI) calculated as weight (kg)/height^2^ (m^2^). While BMI serves as a surrogate marker for adiposity, additional measurements, such as waist circumference, can enhance the diagnosis of obesity. However, its associated metabolic diseases often progress silently in the early stages, making their detection difficult without more comprehensive diagnostic tools. Conventional metabolic markers, including blood pressure, blood glucose, blood lipids, and uric acid levels, provide valuable information but often fail to capture the full extent of metabolic dysfunction.

The onset of various complications following obesity can vary significantly. “Early diagnosis” in the context of obesity-related metabolic diseases refers to the detection of metabolic dysfunction during the preclinical phase, prior to the onset of clinical symptoms. This includes identifying abnormal biomarker levels before overt disease develops. Research indicates that metabolic dysregulation can occur several years before clinical symptoms manifest, highlighting the importance of timely intervention. For instance, fasting blood glucose tests require an eight-hour fasting period and are influenced by biological variability. Furthermore, their reliance on a single time-point measurement can lead to inconsistencies due to sample instability. While the oral glucose tolerance test (OGTT) offers a more detailed metabolic assessment, it is costly, labor-intensive, and may suffer from poor reproducibility. This test involves multiple blood draws over a two-hour period, making it both invasive and uncomfortable for patients.

Diagnosing metabolic-dysfunction-associated steatotic liver disease (MASLD) and metabolic-dysfunction-associated steatohepatitis (MASH) typically requires a liver biopsy, an invasive procedure with risks such as bleeding and, in rare cases, fatal complications. However, it is important to note that diagnosis can also be made through noninvasive imaging techniques, such as ultrasound, computed tomography (CT), and magnetic resonance imaging (MRI), which can assess liver steatosis and inflammation. While these imaging modalities are valuable tools, biopsy results remain critical for definitive diagnosis, especially in cases where there is uncertainty.

Currently, one in eight people worldwide are living with obesity, and its prevalence continues to rise. With obesity rates continuing to rise globally, the prevalence of obesity-related metabolic diseases is also increasing, placing considerable strain on healthcare systems.

Currently, approximately 16% of the global population, equating to around 160 million individuals, are living with obesity, and its prevalence continues to rise [2]. As of 2022, an estimated 462 million individuals are affected by type 2 diabetes [3], while hypertension is attributable to adiposity in 60–70% of adult cases [4]. Furthermore, the global prevalence of fatty liver disease stands at 38.0% [5]. With obesity rates continuing to rise globally, the prevalence of these obesity-related metabolic diseases is also increasing, placing considerable strain on healthcare systems. These conditions collectively impose substantial healthcare costs; for instance, in 2016, the aggregate medical cost due to obesity among adults in the United States was estimated to be USD 260.6 billion [6]. This growing burden underscores the urgency of developing innovative diagnostic approaches.

Although traditional diagnostic methods are often considered the “gold standard”, their limitations highlight the need for noninvasive and accessible biomarkers. To distinguish early from advanced disease, it is crucial to establish quantitative thresholds for biomarkers. For instance, elevated fasting insulin and abnormal glucose tolerance can indicate early metabolic dysfunction, whereas more severe insulin resistance and hyperglycemia are associated with advanced disease. These biomarkers would offer a more convenient, accurate, and patient-friendly approach to diagnosing obesity-related metabolic diseases. Identifying novel biomarkers and refining assay methods are crucial for the development of sensitive, noninvasive tools, particularly for conditions like diabetes mellitus.

Metabolomics and proteomics are pivotal platforms for discovering novel biomarkers in obesity-related metabolic diseases [7,8]. Metabolomics analyzes metabolites in biological samples, providing insights into the metabolic state and enabling the identification of specific signatures associated with obesity and its comorbidities. Proteomics focuses on the large-scale study of proteins, revealing biomarkers that reflect the physiological processes underlying these diseases. Together, these technologies offer robust frameworks for identifying noninvasive biomarkers, significantly enhancing the diagnostic landscape.

### 1.2. Noninvasive Biomarkers: A Shift from Blood to Urine

Urine has emerged as a valuable source of noninvasive biomarkers, with significant potential for early disease detection and metabolic research. Urine contains more than 3000 chemical compounds and metabolites, including metabolic by-products from sources such as diet, pharmaceuticals, environmental contaminants, endogenous waste, and bacterial activity [9]. While many of these compounds were historically poorly characterized, recent research has uncovered their diagnostic potential. For instance, metabolic profiling of urine samples collected over two 24-h periods identified consistent metabolite excretion patterns associated with adiposity [10].

Urine offers several advantages as a noninvasive biomarker. Unlike blood-based markers, urine metabolites are less affected by sampling time or diurnal fluctuations. Urine is easy to collect, has low biological variability, and provides valuable insights into ongoing metabolic processes, making it an ideal candidate for early disease detection and biomarker research. In fact, the growing recognition of these advantages has led to the increasing use of home urine test kits in clinical practice in recent years [11,12].

To systematically evaluate the current landscape of noninvasive diagnostic studies related to urine analysis in obesity-related metabolic diseases, we established specific inclusion criteria. These criteria included noninvasive diagnostic studies utilizing urine analysis that specifically addressed obesity-related metabolic diseases, published in peer-reviewed journals accessible via Google Scholar and PubMed. Exclusion criteria comprised (1) studies focusing on invasive diagnostic methods; (2) research not specifically related to obesity or its metabolic complications; (3) articles not published in English; (4) studies with a sample size of fewer than 20 participants; and (5) reviews, commentaries, or editorials that did not present original research data.

## 2. Advances in Urinary Biomarker Detection Technologies

Urine is now recognized as a valuable source of biological molecules, including proteins and metabolites. In particular, urinary metabolomics has emerged as a key approach for biomarker discovery, providing insights into disease mechanisms, biological pathways, and etiology [13]. Urinary metabolic profiling captures both endogenous physiological processes and exogenous influences, making it a powerful tool in disease research.

Congenital metabolic disorders provide some of the clearest examples of urinary biomarkers. Specific metabolic by-products can accumulate in urine due to genetic deficiencies, allowing for early disease detection. For instance, distinct urinary odors have been investigated as potential indicators of Alzheimer’s disease [14], while elevated levels of Dolichol-18 are commonly detected in the urine of families affected by pigmentary retinitis [15]. Beyond genetic disorders, disease-driven metabolic alterations also manifest in urine. For example, increased urinary levels of N-acetylputrescine have been associated with colorectal cancer, highlighting its potential role as a diagnostic biomarker [16]. Additionally, environmental exposures can influence urinary metabolite profiles. For example, bisphenol A (BPA), an endocrine-disrupting chemical, can be detected in urine and has been proposed as a potential biomarker for prostate cancer [17].

Beyond metabolomics, urinary proteomics is emerging as a powerful tool for diagnosing and monitoring systemic diseases. Urinary proteome analysis in animal models has revealed early disease-related changes that precede alterations in blood parameters, clinical symptoms, and even light microscopy findings. This suggests that urinary biomarkers hold significant promise for the early detection of human diseases, providing a noninvasive and highly informative diagnostic approach [18]. Human urine contains over 1500 proteins and an even greater number of peptide fragments. Many urinary peptides are generated through the hydrolysis and degradation of proteins, which allows for their direct analysis without the need for prior digestion. This feature significantly enhances their compatibility with mass spectrometry techniques, facilitating more efficient and accurate analyses.

Urinary exosomes (UE), small membrane-bound vesicles secreted by epithelial cells in the kidneys and urinary tract, also hold significant potential as noninvasive biomarkers. These vesicles, typically ranging from 40 to 160 nm in diameter, carry stable proteins and RNAs that are more resistant to degradation than their soluble counterparts in urine [19]. For instance, urinary microRNA profiles exhibit distinct alterations in breast cancer patients, suggesting that specific microRNAs could serve as potential biomarkers for breast cancer screening [20]. Furthermore, urinary exosomal microRNAs have shown potential as biomarkers for kidney fibrosis. Their expression profiles can be utilized clinically for the early detection of kidney fibrosis and obesity-associated chronic kidney disease, offering a noninvasive approach to monitor disease progression [21].

To enhance biomarker discovery and validation, metabolomic studies have increasingly employed advanced analytical techniques such as proton nuclear magnetic resonance (^1^H NMR), gas chromatography–mass spectrometry (GC-MS), and ultra-performance liquid chromatography (UPLC) (Table 1). NMR technology was introduced for chemical analysis in the mid-20th century, although its application in metabolomics has gradually developed over the following decades. GC-MS and liquid chromatography–mass spectrometry (LC-MS) are two techniques that have extensive applications in various fields, such as environmental monitoring, food safety, and clinical analysis. After years of development, they have established mature operational protocols and standardized methods that allow for absolute quantification, leading to their increasing use in the analysis of biological fluids. Microfluidic chip technology is relatively new and enables complex analyses in miniaturized, portable devices.

## 3. Disease-Specific Biomarkers and Applications

### 3.1. Type 2 Diabetes

Type 2 diabetes (T2D) is a long-term metabolic disorder that impairs the body’s ability to regulate blood sugar. It is strongly linked to obesity, as excess fat contributes to insulin resistance, which makes glucose regulation more difficult.

Urinary biomarkers have emerged as promising tools for the early detection of T2D and its microvascular complications, which often develop early in the disease.

Proteins involved in insulin signaling, such as protein kinase cAMP-dependent type II regulatory subunit α, growth factor receptor-bound protein 2, and guanine nucleotide-binding protein G(s), are downregulated in the urine of diabetic patients, correlating with disease progression [22]. In addition to proteins involved in insulin signaling, recent research has highlighted urinary volatile organic compounds (VOCs) as potential diagnostic biomarkers for T2D. These VOCs offer a complementary approach in distinguishing T2D from healthy controls [23].

Phosphoenolpyruvate carboxykinase (PEPCK), a key enzyme in gluconeogenesis, is a promising noninvasive marker for assessing insulin resistance. Elevated PEPCK levels have been observed in individuals with prediabetes and diabetic rats compared to nondiabetic controls [24]. Notably, in human urine exosomes, elevated PEPCK levels and a diminished postprandial suppression of gluconeogenesis have been observed. These findings suggest that urinary exosomal PEPCK could serve as a promising biomarker for early-stage diabetes and insulin resistance.

Tracking myoinositol levels in urine before and after meals has been proposed as a simple, noninvasive screening method for diabetes [25]. Due to its stability, myoinositol offers an easier, more convenient alternative to the traditional OGTT, as it can be collected at home without preservatives, enabling large-scale screening for diabetes or glucose intolerance.

Small urinary peptides have been linked to kidney damage in T2D patients, reinforcing their diagnostic value. CHU et al. utilized weak cationic-exchange beads in combination with matrix-assisted laser desorption ionization time-of-flight mass spectrometry (MALDI-TOF MS) to analyze urine peptidome profiles, identifying several proteins, including histidine triad nucleotide-binding protein 1, bifunctional aminoacyl-tRNA synthetase, and clusterin precursor protein as biomarkers capable of distinguishing T2D patients from healthy controls [26]. These noninvasive biomarkers provide a valuable approach for monitoring T2D, particularly in patients without hypertension or hyperlipidemia.

In diabetic kidney disease (DKD), urine serves as a direct indicator of disease severity. Specific urinary markers, such as albumin and sodium excretion, are crucial for detecting T2D and its microvascular complications [27]. These renal tubular biomarkers hold significant potential for early detection and monitoring of renal injury in diabetes, enabling timely intervention to protect kidney function and improve patient outcomes. Simultaneous analysis of urinary biomarkers, including vitamin-D-binding protein (VDBP), retinol-binding protein 4 (RBP4), kidney injury molecule-1 (KIM-1), and tumor necrosis factor receptor-2 (TNFR-2), using Luminex liquid suspension chip technology has demonstrated high diagnostic accuracy, reflecting distinct kidney damage mechanisms [28]. MicroRNAs, such as microRNA 126 and microRNA 770, are emerging as promising biomarkers for diabetic nephropathy. MicroRNA 126, primarily expressed in endothelial cells, is significantly associated with vascular complications in diabetic nephropathy, while microRNA 770, which regulates gene expression involved in cell proliferation and metabolic diseases, may serve as a therapeutic target for managing the disease. Both microRNAs show potential as noninvasive biomarkers for early detection and monitoring of diabetic nephropathy [29]. Integrating urinary biomarkers with advanced diagnostic tools, such as continuous glucose monitoring (CGM) and wearable health devices, could enhance diabetes management by providing a more comprehensive assessment of disease progression.

### 3.2. Hyperlipidemia and MASLD

Urinary biomarkers have demonstrated significant potential in evaluating hyperlipidemia (HLP) and MASLD. Both hyperlipidemia (HLP) and MASLD are closely associated with obesity, as excess body fat promotes lipid imbalances and disrupts lipid metabolism, contributing to these conditions.

Elevated lipid levels and impaired fat metabolism can lead to excessive circulating fatty acids, surpassing the albumin-binding capacity. Researchers have identified 22 potential urinary biomarkers associated with HLP, involving metabolic pathways related to amino acids, fatty acids, nucleotides, steroid hormones, and gut microbiota [30]. These pathways are primarily enriched in alanine, aspartate, and glutamate metabolism; arginine and proline metabolism; lysine degradation; and phenylalanine metabolism, all of which play crucial roles in hyperlipidemia, particularly through their influence on inflammatory responses and oxidative stress.

Notably, lipid oversupply in diet-induced hyperlipidemic rat models resulted in elevated levels of free nucleosides, including deoxyadenosine, uridine, and 3-methyluridine in urine, given that these metabolites serve as fundamental structural units for nucleic acids [31,32]. This suggests that excessive fatty acid circulation may impair DNA and RNA synthesis by degrading nucleic acids, a disruption that could serve as a potential biomarker for lipid-related metabolic disturbances [31]. In this study, lipid oversupply led to significant alterations in endogenous metabolites, and integrating serum and urinary biomarker analysis provided complementary insights into metabolic disturbances associated with hyperlipidemia. In addition, diet-induced hyperlipidemic rats showed increased urinary tryptophan and decreased urinary phenylalanine levels. These alterations may serve as valuable biomarkers for monitoring hyperlipidemia and related conditions, such as atherosclerosis [33].

Furthermore, a population-based study in the U.S. found that elevated BPA levels were associated with an increased risk of all-cause mortality in individuals with hyperlipidemia. This highlights the potential role of environmental factors, such as BPA exposure, in exacerbating hyperlipidemia and underscores the need for further research into noninvasive urinary biomarkers for risk assessment [34].

Urinary biomarkers have also shown promise in diagnosing MASLD. Like hyperlipidemia, MASLD is associated with metabolic disturbances that can be monitored through urinary biomarkers. Liu et al. identified significant alterations in urinary molecular profiles, highlighting dysregulation in carbohydrate metabolism, glycosaminoglycan catabolism, insulin-like growth factor receptor levels, inflammatory responses, the PI3K–Akt signaling pathway, and cholesterol metabolism. Dong et al. identified 31 urinary metabolites differentiating MASLD from MASH, primarily involving nucleic acids and amino acids associated with energy metabolism, amino acid metabolism, and the pentose phosphate pathway [35]. Key metabolites, such as 3-indoleacetic acid, L-carnitine, pyroglutamic acid, and indolelactic acid, proved effective in distinguishing MASH from healthy controls and assessing therapeutic responses. Proteins such as orosomucoid-1 (ORM1) and ceruloplasmin have been identified as potential biomarkers capable of distinguishing mild steatosis from healthy individuals and differentiating severe steatosis from mild cases in MASLD patients [36].

Urinary steroid metabolites have also shown promise in distinguishing MASH-related cirrhosis from alcohol-induced cirrhosis. Moolla et al. demonstrated that a panel of 10 urinary steroid metabolites could achieve an area under the curve (AUC) of 0.83 for this differentiation, providing strong diagnostic value [37]. A combined model of these metabolites, age, and BMI demonstrated even greater diagnostic accuracy, with AUCs of 0.92 and 0.90 for detecting advanced fibrosis and F4 fibrosis (the most advanced stage of fibrosis, indicating cirrhosis), respectively. On the other hand, Haam et al. found that high levels of ethylmalonate, β-hydroxybutyrate, and sulfate were significantly related to a low probability of hepatic fibrosis [38]. As these metabolites were not influenced by obesity or insulin resistance, their deficiency in urine may serve as an early indicator of hepatic fibrosis progression.

Urinary metabolic profiling has also been explored as a tool for monitoring treatment responses in MASLD. In children receiving probiotic medical food, metabolic alterations were observed in amino acid metabolism (valine and tyrosine), nucleic acid degradation (pseudouridine), and gut microbial metabolism (2-hydroxyisobutyrate from valine degradation) [39]. These findings highlight the potential of urinary biomarkers to serve as noninvasive, effective alternatives for diagnosing and managing liver diseases.

### 3.3. Hyperuricemia

Hyperuricemia, characterized by elevated blood uric acid levels, arises from disruptions in uric acid production, metabolism, and excretion. It is increasingly recognized as a metabolic disorder associated with obesity, as excess body fat contributes to insulin resistance, reduced renal uric acid excretion, and heightened systemic inflammation—factors that collectively drive uric acid accumulation. While blood uric acid remains the primary diagnostic marker, urinary uric acid levels provide critical insights into renal excretion dynamics, offering a more comprehensive assessment of metabolic imbalances.

Metabolomic analysis has identified key metabolic alterations in gout and hyperuricemia. Notably, intermediates of the citrate cycle, particularly 2-ketoglutarate, were significantly elevated in gout patients, suggesting disruptions in energy metabolism associated with urate accumulation. Additionally, urinary nicotinate emerged as a critical biomarker, exhibiting a fold difference of 6.515 between hyperuricemic and healthy individuals [40]. This finding underscores the link between gout and the accumulation of monocarboxylates, such as lactate and nicotinate, which are excreted through renal pathways coupled with urate reabsorption.

Proteomic profiling of 2119 proteins revealed significant changes in hyperuricemia, with 11 proteins downregulated and 2 upregulated in affected individuals [41]. Among the downregulated proteins, V-type proton ATPase subunit B 1 (VATB1) and complement factor D (CFAD) are associated with insulin resistance and the regulation of insulin secretion, while Apolipoprotein C-III (APOC3), a key regulator of triglyceride metabolism, was significantly reduced, suggesting its potential as a marker for monitoring hyperuricemia-related metabolic complications. These findings offer promising avenues for early detection and timely intervention, emphasizing the role of urinary proteomics in refining diagnostic and therapeutic strategies for hyperuricemia.

### 3.4. Hypertension

Hypertension, or high blood pressure, is a prevalent condition commonly associated with obesity. Excess body fat contributes to increased blood pressure by raising blood volume, cardiac output, and vascular resistance. Given the ease of blood pressure measurement, developing methods to predict the onset of hypertension or monitor its progression holds significant clinical value.

Hypertensive patients exhibit distinct metabolic profiles, and identifying these changes can offer valuable insights into the markers and pathways involved in hypertension development. Metabolomic analyses comparing normotensive, prehypertensive, and hypertensive individuals have identified several metabolites—stearidonate, hexadecadienoate, N6-carbamoylthreonyladenosine, 9- and 13-S-hydroxyoctadecadienoic acid (HODE), 2,3-dihydroxy-5-methylthio-4-pentenoate (DMTPA), and linolenate—that are strongly associated with an elevated risk of hypertension. These metabolites show significant diagnostic potential, with AUC values exceeding 0.7 in both discovery and validation cohorts [42], and can predict the progression from prehypertension to hypertension.

Urinary metabolites also offer predictive markers for hypertensive progression [43]. The urinary albumin-to-creatinine ratio independently predicts future increases in systolic blood pressure among the general Japanese population, underscoring its significance as an early indicator of rising blood pressure [44]. Moreover, elevated urinary levels of epidermal growth factor (EGF) have been linked to a reduced risk of hypertension over a 10-year period in relatively healthy middle-aged individuals [45]. Given EGF’s important role in repairing kidney tubule damage [46], urine concentrations of EGF may serve as a marker for the kidney’s tubule reparative capacity. Unlike most urine biomarkers, higher urine EGF is considered protective, correlating with lower markers of tubular damage.

In addition, a meta-analysis identified a positive association between urinary polycyclic aromatic hydrocarbon (PAH) metabolites and hypertension risk [47]. While most PAHs are rapidly excreted in urine after exposure, providing limited insight into long-term exposure, urinary PAH measurements offer a noninvasive, real-time method to monitor short-term environmental influences on hypertension risk. Despite the limitations, these measurements still provide valuable information on the immediate impact of environmental factors such as air pollution.

### 3.5. Polycystic Ovarian Syndrome

Polycystic ovarian syndrome (PCOS) is a prevalent endocrine disorder affecting reproductive-aged women, characterized by irregular menstruation, hyperandrogenism, and polycystic ovaries. Its pathogenesis is multifactorial, involving insulin resistance, low-grade inflammation, genetic predisposition, and excess androgen production. Obesity frequently coexists with PCOS, exacerbating insulin resistance and hormonal imbalances, thereby worsening metabolic complications.

Recently, several urinary biomarkers have emerged as potential indicators of these metabolic and hormonal changes, offering new possibilities for early detection and monitoring of PCOS. Among the differentially expressed metabolites, four differentially expressed metabolites—stearic acid, palmitic acid, benzoylglycine, and threonine—have been proposed as potential biomarkers of PCOS pathogenesis. Notably, fatty acids such as palmitic and stearic acid, which have not traditionally been associated with PCOS, demonstrate strong diagnostic potential (AUC > 0.99) [48], indicating that metabolic abnormalities in PCOS extend beyond hormonal imbalances.

Further metabolomic analyses identified two novel urinary biomarkers—testosterone-glucuronide and 11α-hydroxyprogesterone—along with four additional candidate biomarkers: benzofenap, methionyl-phenylalanine, Glycerolipid(18:4(6Z,9Z,12Z,15Z)/0:0/0:0), and 2-(14,15-epoxyeicosatrienoyl) glycerol, all specifically detected in PCOS patients [49]. Unlike plasma testosterone, which is subject to circadian rhythm variations, urinary testosterone-glucuronide concentrations remain stable, making it a reliable indicator of androgen production and a potential biomarker for PCOS diagnosis and monitoring.

In addition to hormonal and metabolic markers, urinary metabolites linked to insulin resistance have been explored in PCOS patients. Research by Di Egidio et al. identified 3-phenylpropionate and pyruvate as rapid and reliable indicators of insulin sensitivity in nondiabetic women with PCOS [50]. 3-phenylpropionate, a metabolite derived from gut microbial metabolism, reflects gut microbiota alterations associated with PCOS and insulin resistance. Pyruvic acid, the end product of glycolysis, plays a crucial role in cellular metabolism, and its dysregulation is well documented in individuals with PCOS and insulin resistance [51]. These findings underscore the interplay between gut microbiota, energy metabolism, and insulin sensitivity, reinforcing the potential of urinary metabolomics in assessing metabolic health in PCOS.

### 3.6. Metabolic Syndrome

Metabolic syndrome (MetS) is a cluster of metabolic abnormalities closely linked to obesity, encompassing insulin resistance, hypertension, dyslipidemia, and central obesity. Obesity contributes to MetS by exacerbating insulin resistance and disrupting lipid metabolism. It is estimated that between 30% and 50% of individuals with obesity develop MetS globally.

Recent research has identified several urinary biomarkers that provide valuable insights into its diagnosis and progression. Individuals with MetS exhibit elevated levels of metabolites such as pyruvate, α-ketoglutarate, α-ketoisovalerate, α-ketoisocaproate, formiminoglutamate, and quinolinate, with odds ratios ranging from 1.915 to 2.809 in logistic models adjusted for age and sex [52]. These biomarkers are strongly linked to disrupted metabolic pathways, including impaired insulin signaling and oxidative stress. Furthermore, increased quinolinate levels—a neurotoxic metabolite in the kynurenine pathway—suggest a role for systemic inflammation and redox imbalance in the pathogenesis of MetS.

As MetS progresses, the urine metabolome undergoes continuous and monotonic changes, with significant upregulation or downregulation of 17 key metabolites, including glucose, lipids, aromatic amino acids, salicyluric acid, maltitol, trimethylamine N-oxide, and p-cresol sulfate. These metabolic alterations have been integrated into predictive models, achieving AUC values between 0.83 and 0.87, highlighting their potential for identifying individuals with MetS [53].

## 4. Challenges and Future Perspectives

Noninvasive urinary biomarkers offer significant potential for diagnosing obesity-related metabolic disorders (Table 2); however, several challenges must be addressed before their widespread clinical adoption. A major hurdle lies in the variability of detection methods and their sensitivity. Many current assays lack standardization, resulting in inconsistencies across analytical platforms [54]. Most tests rely on fasting morning urine samples, but this approach may be unreliable for individuals with impaired renal function, as altered biomarker concentrations can skew results [55]. Standardized urine collection protocols—such as first-morning versus random sampling—are essential to ensure comparability across studies.

In addition, the application of metabolomics in multi-center studies is on the rise; however, individual factors such as diet, age, and sex significantly influence urinary metabolite levels. Thus, the evaluation of geographical factors, including dietary habits, should be carefully considered in the interpretation of metabolomic data from these studies. Research has shown that the geographical location of populations exerts a significant impact on urinary ^1^H NMR metabolomic profiles, primarily due to variations in dietary intake [56]. Consequently, it is recommended that diet standardization be implemented for several days prior to a dietary intervention to minimize intra- and inter-subject variability [57].

Moreover, stable genetic and environmental factors account for approximately 47% of the variation observed in urinary metabolic profiles [58]. Using targeted metabolomics, the effects of gender, diurnal variation, and age on human urinary metabolomic profiles are also evident [59]. Notably, metabolites related to mitochondrial energy metabolism have been identified as differentiators between genders and age groups. Additionally, dietary components and certain metabolites associated with circadian rhythms have been found to influence the differentiation of urine collected at various times of the day.

Research examining the urine of normal children and adults has revealed that the metabolic characteristics for each age group can reflect metabolic status during different life stages and may contribute to the incidence of age-dependent diseases. Specifically, the pantothenate and CoA biosynthesis pathways, as well as alanine metabolism, were enriched in early life. In contrast, androgen and estrogen metabolism exhibited heightened activity during adolescence and young adulthood. Furthermore, pyrimidine metabolism was found to be enriched in the geriatric stage. It is also noteworthy that sex-dependent urinary metabolites are significantly more pronounced in adults than in children [60]. Understanding the mechanisms behind these differences will be crucial for the development of new diagnostic tests.

Furthermore, biomarker validation must align with established regulatory frameworks, such as the FDA’s Biomarker Qualification Program, which mandates rigorous analytical and clinical validation as well as qualification for specific contexts of use.

Bridging the gap between biomarker research and clinical practice presents another key challenge. While individual biomarkers or biomarker panels can differentiate diseases and provide insight into molecular mechanisms, their clinical translation involves three critical stages: biomarker validation, diagnostic kit development, and integration into clinical guidelines. Each stage introduces technical and regulatory complexities, including the need for comprehensive validation to confirm diagnostic accuracy, safety, and reproducibility. Regulatory approval requires extensive clinical trials and supporting data to demonstrate efficacy. In addition, financial considerations, such as reimbursement models, must be addressed to ensure accessibility within healthcare systems. Overcoming these challenges is essential for establishing noninvasive urinary biomarkers as reliable diagnostic tools in routine clinical practice.

Artificial intelligence (AI) and multi-omics approaches have the potential to transform noninvasive diagnostics by integrating urinary biomarkers with clinical parameters to enhance accuracy and disease characterization [8]. AI-driven models can analyze large-scale metabolomics and proteomics datasets, uncovering hidden patterns in complex disease pathways. Real-world applications already leverage AI to refine disease classification, predict progression, and identify novel biomarkers. However, before widespread clinical implementation, these tools require extensive validation across diverse populations to ensure robustness and generalizability.

A critical challenge in AI adoption is model interpretability. Clinicians must be able to understand how AI systems generate predictions to ensure transparency in medical decision making. In addition, AI models must integrate high-quality, standardized data while minimizing biases that could distort clinical insights. Addressing these concerns is crucial for AI to become a reliable tool in managing obesity-related metabolic disorders. Beyond AI, standardizing urinary biomarker detection methods—such as urine sampling protocols—and conducting multi-center validation trials remain key priorities. Future research should focus on AI-driven multi-omics integration while aligning with regulatory frameworks, such as the FDA Biomarker Qualification Program, to facilitate clinical translation. Ultimately, the successful adoption of AI in noninvasive diagnostics will depend on overcoming challenges related to data quality, regulatory approval, and interpretability.

## 5. Conclusions

Obesity-related metabolic diseases are complex and often insidious in onset, challenging the effectiveness of traditional diagnostic markers. Urine, as a metabolically rich and easily accessible biofluid, offers unique advantages for early detection. Emerging advances in urine metabolomics and proteomics have illuminated novel biomarkers that better reflect the intricate nature of metabolic dysfunction. Moving forward, the integration of standardized methodologies, robust clinical validation, and artificial intelligence-driven multi-omics analysis will be pivotal. Harnessing these innovations holds the key to transforming early diagnosis and enabling more precise, personalized management of obesity-related diseases.

## Figures and Tables

**Table 1:** Noninvasive urinary biomarker detection technologies for metabolic diseases.

Technology	Abbreviation	Comparative Cost Tier	Advantages	Limitations	Primary Applications
Nuclear Magnetic Resonance	NMR	$$	- Provides detailed information on molecular structures - Can analyze complex mixtures - Enables dynamic tracking of chemical reactions	- Low sensitivity for low-abundance metabolites - Requires expensive equipment - Requires skilled operators - Requires isotope enrichment (e.g., carbon-13) to improve sensitivity - Long analysis time for multi-dimensional experiments - High maintenance costs (e.g., liquid helium cooling)	- Structural biology - Metabolomics - Drug discovery - Material science
Gas Chromatography–Mass Spectrometry	GC-MS	$$$	- High sensitivity and specificity - Suitable for volatile compounds - Reliable - Compatible with standard compound databases for rapid identification - Ideal for environmental pollutants	- Requires sample derivatization - High operational cost - Complex sample preparation needed - Unsuitable for large or thermally unstable molecules (e.g., proteins) - Limited column lifespan - Time-consuming derivatization for polar/nonvolatile compounds	- Environmental monitoring - Forensic analysis - Food safety testing - Petrochemical analysis
Liquid Chromatography–Mass Spectrometry	LC-MS	$$$	- Excellent for analyzing complex mixtures - High sensitivity and accuracy - Suitable for both targeted and untargeted analysis - Widely used in metabolomics and drug discovery	- Expensive - Requires skilled operators - Ion suppression may be observed in complex samples - High maintenance costs for hyphenated systems - Time-consuming method development - Reliant on MS compatibility	- Pharmaceutical R&D - Clinical toxicology - Metabolomics - Proteomics
Matrix-Assisted Laser Desorption/Ionization Time-of-Flight Mass Spectrometry	MALDI-TOF	$$$$	- High sensitivity for large biomolecules - Fast analysis time - Suitable for high-throughput applications - Minimal sample preparation for intact protein analysis - Allows direct tissue section analysis	- Limited ability to analyze small molecules - Sample preparation can be challenging - Requires expensive instruments - Requires matrix optimization for reproducibility - Limited quantitative accuracy - Matrix interference in low-mass regions (<500 Da)	- Clinical microbiology (pathogen identification) - Proteomics - Biomarker discovery - Tissue imaging
Ultra Performance Liquid Chromatography	UPLC	$$	- High resolution and precision - Faster than traditional HPLC - Suitable for complex mixtures - Reduced solvent consumption (eco-friendly)	- Requires expensive instruments - Requires highly trained personnel - Durable components are required for high-pressure systems - Strict sample filtration requirements - Limited qualitative analysis capability - Often paired with MS for qualitative analysis	- Pharmaceutical analysis - Environmental analysis - Food and beverage testing - Metabolomics
Proteomics (Mass-Spectrometry-based)	Proteomics (MS-based)	$$$$	- Provides in-depth insights into protein expression and modifications - High throughput - Quantifies low-abundance proteins - Detects post-translational modifications (e.g., phosphorylation) - Supports multiplexed quantification (e.g., TMT and iTRAQ)	- High equipment and operational costs - Complex sample preparation - Requires specialized expertise - Limited dynamic range (high-abundance proteins may mask low-abundance signals) - Massive data storage/processing demands	- Biomarker discovery - Drug target identification - Clinical proteomics - Systems biology
Microfluidic Chips	Microfluidic Chips	$$$	- Miniaturized, portable, and cost-effective - Potential for point-of-care diagnostics - Requires smaller sample volumes - Enables integrated workflows (separation + detection on-chip) - High-throughput parallel processing	- Limited sensitivity compared to larger instruments - Still under development for widespread clinical use - Custom chip designs limit versatility - Long-term stability issues (e.g., clogging) - Complex manufacturing processes	- Point-of-care diagnostics - Single-cell analysis - Lab-on-a-chip applications - Environmental monitoring
Portable Devices (point-of-care diagnostics)	POCT	$/$$	- Fast, real-time results - User-friendly and easy to operate - Suitable for remote or emergency settings - Supports wireless data transfer (e.g., smartphone integration)	- Limited sensitivity and accuracy - Often less comprehensive than lab-based technologies - Frequent calibration needed (sensitive to environmental conditions) - Limited battery life - Narrow target scope (single/few analytes)	- Emergency medicine - Remote health monitoring - Field diagnostics - Wearable health devices

Cost Tier: $ = Cheap, $$ = Moderate, $$$ = Expensive, $$$$ = Very Expensive; Abbreviations: VOCs, Volatile Organic Compounds. TMT, Tandem Mass Tags. iTRAQ, Isobaric Tags for Relative and Absolute Quantification.

**Table 2:** Summary of studies investigating potential biomarkers for obesity-related metabolic diseases.

Year	Disease	Population	Results	Reference
2023	T2D	52 T2D, 53 controls	- AUC: 0.771 for protein kinase CAMP-dependent type II regulatory subunit α (PRKAR2A) - AUC: 0.751 for growth factor receptor bound protein 2 (GRB2) - AUC: 0.739 for guanine nucleotide-binding protein G(s) (GNAS)	[22]
2018	T2D	73 T2D, 67 controls	- AUC: 0.88 analyzed via Field-Asymmetric Ion Mobility Spectrometry - AUC: 0.85 analyzed via FOX4000	[23]
2024	Renal injury in diabetes mellitus	285 with renal injury, 122 without renal injury	- Renal tubule biomarkers (N-acetyl-β-D-glucosaminidase/Cr) may be used as an indicator in the early detection and monitoring of renal injury in diabetes mellitus.	[27]
2020	T2D	22 T2D, 93 normal	- AUC: 0.83 for UMI 2h-postprandial - AUC: 0.82 for ΔUMI (UMI 2h-postprandial minus UMI premeal). UMI, urinary myoinositol.	[25]
2013	T2D	28 T2D, 29 controls	- Histidine triad nucleotide-binding protein 1, bifunctional aminoacyl-tRNA synthetase, and clusterin precursor protein	[26]
2024	Diabetic kidney disease	585 T2D, 152 with DKD	- AUC: 0.780 for vitamin D binding protein (VDBP) - AUC: 0.812 for a combination of VDBP, retinol-binding protein4 (RBP4), kidney injury molecule-1 (KIM-1), and tumor necrosis factor receptor-2 (TNFR-2)	[28]
2022	MASLD	10 controls, 10 mild hepatic steatosis patients, and 10 severe hepatic steatosis patients	For distinguishing between mild steatosis from controls: - AUC: 0.78 for ceruloplasmin - AUC: 0.87 for alpha-1-acid glycoprotein 1 (ORM1) For distinguishing between severe steatosis from mild steatosis: - AUC: 0.79 for ceruloplasmin - AUC: 0.81 for ORM1	[36]
2017	MASLD	33 MASLD, 45 MASH, and 30 controls	For distinguishing MASH from controls: - AUC: 0.82275 for indolelactic acid - AUC: 0.80159 for gluconic acid For distinguishing MASH from MASLD: - AUC: 0.65087 for pyroglutamic acid	[35]
2022	MASLD	Hepatic steatosis and fibrosis in 68 men and 65 women	- High formiminoglutamate and phydroxyphenyllactate levels were positively associated with hepatic steatosis - High sulfate, ethylmalonate, and β-hydroxybutyrate levels were significantly associated with lower hepatic fibrosis probability	[38]
2019	MASLD	121 with biopsy-proven MASLD, 48 with alcohol-related cirrhosis, and 106 controls	For distinguishing advanced (F3-F4) from early (F0-F2) fibrosis: - AUC: 0.92 by urinary steroid metabolome For distinguishing patients with advanced fibrotic MASLD from controls: - AUC: 0.99 by urinary steroid metabolome For distinguishing patients with cirrhosis from controls: - AUC: 1.0 by urinary steroid metabolome For distinguishing MASLD cirrhosis from alcohol-related cirrhosis: - AUC: 0.83 by urinary steroid metabolome	[37]
2024	Hyperuricemia	26 HUA patients, 25 controls	- 11 proteins decreased and 2 proteins increased in HUA samples	[41]
2016	Hypertension	118 patients, 30 non-hypertensive subjects	For predicting albuminuria development: - AUC: 0.942 for combination of guanidinoacetate, pantothenate, 3-ureidopropionate, oxaloacetate, and pyruvate - AUC: 0.892 for combination of 3-hydroxybutyrate and pyruvate - AUC: 0.861 for combination of guanidinoacetate, glutamate, and pantothenate	[43]
2024	Hypertension	72 pre-hypertensive participants, 72 controls	- AUC: 0.7365 for six metabolites—stearidonate (18:4n3), hexadecadienoate (16:2n6), N6-carbamoylthreonyladenosine, 13-HODE + 9-HODE, 2,3-dihydroxy-5-methylthio-4-pentenoate (DMTPA), and linolenate [alpha or gamma; (18:3n3 or 6)] - AUC: 0.712 for 13-HODE + 9-HODE and DMTPA	[42]
2018	PCOS	21 PCOS, 16 controls	- AUC: 0.947 for stearic acid - AUC: 0.991 for salmitic acid - AUC: 0.905 for benzoylglycine - AUC: 0.941 for threonine	[48]
2021	PCOS	18 with less insulin secretion, 24 with more insulin secretion	- AUC: 0.775 for 3-phenylpropionate - AUC: 0.851 for pyruvate	[50]
2021	MetS	11,754 individuals, 4–5% with developed MetS	- AUC: 0.83–0.87 for metabolic signatures	[53]

## Data Availability

Not applicable.
